# Identification of a non-classical three-dimensional nuclear localization signal in the intestinal fatty acid binding protein

**DOI:** 10.1371/journal.pone.0242312

**Published:** 2020-11-12

**Authors:** Mariana Suárez, Lucía Canclini, Adriana Esteves

**Affiliations:** 1 Sección Bioquímica, Facultad de Ciencias, Universidad de la República, Montevideo, Uruguay; 2 Departamento de Genética, Instituto de Investigaciones Biológicas Clemente Estable, Montevideo, Uruguay; Louisiana State University Health Sciences Center, UNITED STATES

## Abstract

The intestinal fatty acid binding protein (FABP) is a small protein expressed along the small intestine that bind long-chain fatty acids and other hydrophobic ligands. Several lines of evidence suggest that, once in the nucleus, it interacts with nuclear receptors, activating them and thus transferring the bound ligand into the nucleus. Previous work by our group suggests that FABP2 would participate in the cytoplasm-nucleus translocation of fatty acids. Because the consensus NLS is absent in the sequence of FABP2, we propose that a 3D signal could be responsible for its nuclear translocation. The results obtained by transfection assays of recombinant wild type and mutated forms of *Danio rerio* Fabp2 in Caco-2 cell cultures, showed that lysine 17, arginine 29 and lysine 30 residues, which are located in the helix-turn-helix region, would constitute a functional non-classical three-dimensional NLS.

## 1. Introduction

Fatty acid binding proteins (FABPs) belong to the intracellular lipid binding proteins (iLPs) [1]. FABPs were described for the first time by Ockner and co-workers as intracellular cytosolic proteins with a relatively low molecular mass (14–15 kDa), able to bind fatty acids and other lipid ligands [2]. Up to now, nine members have been found in mammals and eleven in non-mammals, and have been named according to the tissue where they were first identified [3]. Despite there being a variable amino acid sequence identity among the different FABP members, these proteins fold in a highly conserved tertiary structure [4]. A typical member of the family consists of 127–134 amino acid residues with ten antiparallel β-strands folded into a barrel capped by two short α-helices [5].

The major role of these proteins is to facilitate the entry of hydrophobic molecules (mainly fatty acids (FAs), but also endocannabinoids and lipophilic drugs) into cells [6–10]. Moreover, FABPs could also be involved in the intracellular transport and compartmentalization of the ligands, as well as in the removal of fatty acids from intracellular depots [11–13]. Members of this family of proteins might exert tissue-specific functions and regulatory functions outside their tissue of expression [14, 15]. They are also bio-markers of tissue injury, metabolic disorders and diseases, and could potentially be used as therapeutic targets [14, 16–20]. However, the mechanisms by which these proteins act and the precise *in vivo* function of each FABP remain to be clarified.

The dietary fat is the major source of lipids found in the intestinal lumen. It is mainly constituted by triglycerides. Lipid hydrolysis releases great quantities of long-chain fatty acids absorbed by the enterocytes under complex mechanisms, involving both a passive diffusion and a protein-mediated transport [6, 21–25]. Once in the cell, dietary FAs are reversibly bound to lipid-binding proteins. The intestinal mucosa and its enterocytes provide a very attractive system for evaluating the fate of exogenous FAs inside cells. FABP1, FABP2 and FABP6 are the most strongly expressed FABP family members in the human intestine and these proteins were found in abundance in absorptive cells [26–28]. Zebrafish is a useful model to study intestinal FABP function, in particular drFabp2, which is highly expressed in the anterior intestine and shares 90% similarity with the human counterpart [29, 30].

FABPs may channel FAs and other lipophilic ligands into nuclei, transferring them to nuclear receptors which in turn modulate transcriptional activity [8, 30–36]. Using zebrafish as an in vivo model, it has been demonstrated that drFabp2 is involved in fatty acid nuclear translocation [30]. Conventional protein import mechanism into the nucleus implies the presence of Nuclear Localization Signals (NLS) that direct the protein to pass through the nuclear pores. NLS in iLP family members have been described for hCRABPII, mFABP4 and hFABP5 as a tertiary structure localization signal conformed by three amino acids in the two α-helices [35, 37–39]. Our interest in the mechanism of nuclear internalization of FABP2 led us to search the NLS of *Danio rerio* Fabp2 using directed mutagenesis and in vivo subcellular localization analysis on Caco-2 cells.

## 2. Materials and methods

### 2.1. FABPs structural analysis

The following sequences were aligned using Clustal Omega [40]: hCRABP-2 (CAG29353.1); hFABP4 (CAG33184.1); hFABP5 (NP_001435); hFABP2 (CAG33184.1) and drFabp2 (AAF00925.1). Structural analysis was performed using human apo FABP2 (PDB ID: 1KZW), human apo FABP4 (PDB ID: 3RZY), human holo FABP2 bound to oleic acid (PDB ID: 2MO5), mouse holo FABP4 bound to oleic acid (PDB ID: 1LID) and linoleic acid (PDB ID: 2Q9S). Images and structure overlays were generated in Swiss-PdbViewer 4.1.0 software [41].

### 2.2. Directed mutagenesis and subcloning

*Danio rerio* fapb2 full-length coding sequence cloned into pET5a+ plasmid was used as a template for all drfabp2 mutagenesis reactions [30]. drFabp2 mutants were generated by two rounds of site-specific PCR mutagenesis with the QuickChange II Site-Directed Mutagenesis kit (Agilent Technologies), using the following primers:

- 5’-GCACGCAATGAGAACTACGAGGCCTTCATGGAACAAATGGGCGTC-3’ and 5’-GACGCCCATTTGTTCCATGAAGGCCTCGTAGTTCTCATTGCGGTC-3’, for the lysine 17 to alanine 17 mutation;
- 5’-GAACAAATGGGCGTCAACATGGTGAAAGCCGCCCTGGCTGCCCATGACAACCTG-3’ and 5’-CAGGTTGTCATGGGCAGCCAGGGCGGCTTTCACCATGTTGACGCCCATTTGTTC-3’, for the lysine 28 and arginine 29 to alanine 28 and 29 mutations; and
- 5’-GAACAAATGGGCGTCAACATGGTGGCCGCCAAACTGGCTGCCCATGACAACCTG-3’, and 5’-CAGGTTGTCATGGGCAGCCAGTTTGGCGGCCACCATGTTGACGCCCATTTGTTC-3’ for the arginine 29 and lysine 30 to alanine 29 and 30 mutations.

For subcloning into the mammalian expression vector, full-length wild-type and mutated drfabp2 were amplified by PCR with 5’- CCGGGATCCATGACCTTCAACGGGACCTGG-3’ (sense) and 5’-CCGCTCGAGGCCGGCGCCGGCGCCGGCGCCGGCAGCCCTCTTGAAAATCCTCT-3’ (antisense) primers in order to add the BamHI/XhoI restriction enzyme sites, as well as the additional linker sequence of GCCGGC repeats. After amplification, PCR generated fragments were subcloned into the mammalian expression vector pCDNA-EGFP, previously digested with the mentioned restriction enzymes. Plasmids were purified using PureLink HiPure Plasmid Midiprep Kit (Invitrogen).

### 2.3. Bacterial recombinant protein expression and purification

Full-length wild-type and mutated *Danio rerio* fabp2 cloned into the pET5a+ vector were transformed into *Escherichia coli* strain BL21 (DE3) cells. Cultures (250 mL) were grown using ZYM5052 broth supplemented with ampicillin (100 mg/mL) at 37°C for 20h. Cells were collected by centrifugation at 5500 xg for 10 min and lysed by sonication in lysis buffer (30 mM Tris-HCl pH 8.3, 500 mM NaCl, 1 mM DTT, 5 mM MgCl2, 1 mM phenylmethanesulfonyl fluoride). Soluble fractions were separated by centrifugation at 12000 xg for 15 min at 4°C. Recombinant proteins were purified by precipitation with two consecutive rounds of ammonium sulfate fractionation (30% and 50%). The supernatants from the last ammonium sulfate precipitation were submitted to gel filtration chromatography (HiPrep 26/60 Sephacryl S-100 HR, GE Healthcare). drFabp2-containing fractions were collected and then submitted to ion-exchange chromatography (HiTrap 5 mL Q XL, GE Healthcare). Fractions collected from ion-exchange chromatography experiments were pulled and delipidated using Lipidex (Hidroxyalkoxipropil-Dextran, Type VI, Sigma H6258) in order to generate apo-Fapb2. Each step of purification was evaluated using SDS-PAGE.

### 2.4. Circular dichroism

Measurements were carried out at 20°C on a Jasco 810 spectropolarimeter (Jasco Corporation, Japan) equipped with a peltier-effect device for temperature control. The instrument was calibrated with (+)-10-camphorsulfonic acid following the manufacturer’s instructions. Scan speed was set to 20 and 50 nm/min (near UV and far UV, respectively) with a 1 sec response time, 0.2 nm data pitch and 1 nm bandwidth. Near-UV measurements were carried out in 1.0 cm cells containing 30 μM protein in 5 mM sodium phosphate pH 7.0, 100 mM NaF. In the far UV, 0.1 cm cells were used and protein concentration was 15 μM in 5 mM sodium phosphate pH 7.0, 100 mM NaF. Five spectra were recorded and averaged for each sample and are presented smoothed with a fourth-degree 10-point moving-window polynomial Savizky-Golay filter.

### 2.5. Binding assays

Wild type and mutated drFabp2 were titrated with BODIPY FL C16 and Kd values were determined by fitting the data to a hyperbolic equation:

$${\text{F}}_{\text{OBS}}={\text{F}}_{\text{MAX}}\times \;\left[\text{BODIPY}\;\text{FL}\;\text{C}16\right]/(\text{Kd}+\left[\text{BODIPY}\;\text{FL}\;\text{C}16\right],$$

where F_OBS_ is the observed (measured) fluorescence intensity at each concentration of BODIPY FL C16 and F_MAX_ is the fluorescence intensity at saturating concentrations of BODIPY FL C16.

Fluorescence emission was measured at an excitation wavelength of 530 nm according to the maximum observed in the BODIPY FL C16 emission spectrum in aqueous solution. Binding assays were performed at 25°C in a Chronos FD Fluorescence Lifetime Spectrometer.

### 2.6. Expression assays in eukaryotic cells

Caco-2 cells (ATTC-HTB-37, Institute Pasteur, Montevideo, Uruguay), were cultured in Dulbecco´s modified Eagle´s medium (DMEM-HG, Capricorn) supplemented with 4.5 mg/mL glucose and 10% fetal bovine serum (Capricorn). 1x10^5^ cells were seeded in 24-well plates, incubated at 37°C, 5% CO_2_ for 24 h and then transfected with 1 μg of plasmid DNA using 1.25 μL of lipofectamine LTX (Invitrogen) according to manufacturer's instructions. At 48 h post-transfection, the cells were fixed with 4% paraformaldehyde in PBS and nuclei were counterstained with DAPI (300 nM, Invitrogen). For the classical nuclear import mechanism inhibition experiments, 40 μM Importazole (MedChemExpress) was added to the medium 24 h before fixation. For the analyses of subcellular localization in response to oleic acid, media was changed to serum-free DMEM 48 h after transfection. This medium was supplemented with 4.5 mg/mL glucose and 10 μM oleic acid-bovine serum albumin (Sigma) and the cells were incubated overnight before fixation. Two replicates of each experimental condition were analyzed.

### 2.7 Immunocytochemistry

Cells were fixed and permeabilized for 15 min in 4% paraformaldehyde in PBS supplemented with 0.5% Triton X-100, rinsed in PBS three times, 5 min each, and incubated with blocking buffer (1% BSA, 0.05% Triton X-100 in PBS) for 30 min at room temperature. Cells were incubated with the anti-c-Myc antibody (1/100, Thermo Fisher Scientific) overnight at 4°C and then incubated with the secondary antibody (1:1000, goat anti-mouse conjugated with Alexa 488; Invitrogen, Thermo Fisher Scientific) for 45 min at room temperature. DAPI (300 nM; Invitrogen) counterstain of nuclei was also performed. Finally, cells were mounted using Prolong Glass Antifade (Invitrogen, Thermo Fisher Scientific).

### 2.8 Confocal microscopy and image processing

For confocal microscopy analyses a Zeiss LSM800 furnished with a 63x oil immersion objective (NA: 1.4, Carl Zeiss International, Oberkochen, Germany) was used. All images were acquired at identical, previously optimized settings, using the same photomultiplier values and low laser power (0.2%). Images were analyzed using Fiji ImageJ [42]. The subcellular localization of each EGFP-tagged drFabp2 was expressed as the ratio of the integrated density of nuclei over the integrated density of cytoplasm. For each experiment, a total of 35 individual cells were analyzed. Shapiro-Wilk test calculator was used to assess data normality. Mean and standard deviation was calculated for each condition. Data was analyzed using Kruskal-Wallis test at a confidence level of 0.01.

## 3. Results

**Fig 1:**
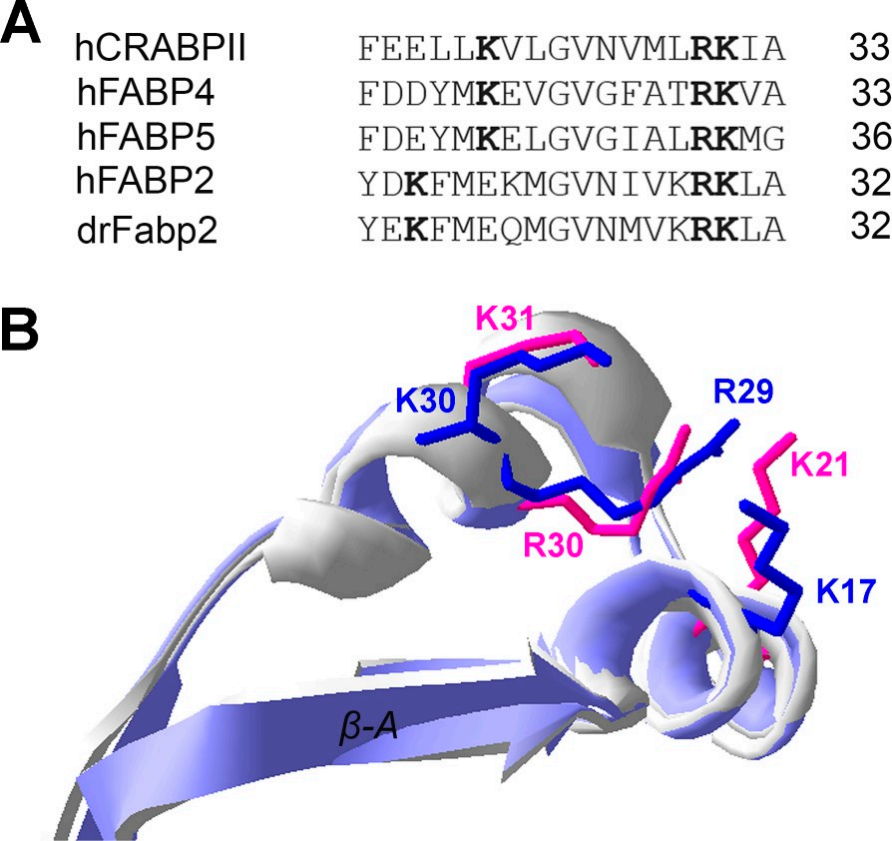
Nuclear localization signal analysis. **A)** α-helical regions of ILPs with described NLS (human CRABPII, FABP4 and FABP5) are shown aligned with human (FABP2) and Danio rerio FABP2 (Fabp2). Basic amino acids that compose the NLS are highlighted in bold. **B)** Superimposed structures of human apoFABP4 (3rzy.pdb) and human apoFABP2 (1kzw.pdb) α-helical region. Side chains of NLS amino acids are shown for human FABP4 (magenta) and human FABP2 (blue).

Due to our interest in the existence of a Nuclear Localization Signal (NLS) in the *Danio rerio* Fabp2 protein, we analyzed the structure of the α-helical region of this protein. The NLS described for hCRABPII, mFABP4 and hFABP5 is composed of a 3D triad of basic amino acids (KRK) [35, 37–39]. The first lysine is located in the α-helix I (position 21 in hFABP4), while the other basic amino acids are two contiguous arginine and lysine residues (positions 30 and 31 in hFABP4) located in the α-helix II (Fig 1A). Sequence alignment of the α-helical region from intestinal FABPs with previously described NLS did not show an exact match (Fig 1A), since the first lysine of the NLS was aligned with a glutamic residue. However, superimposed structures of human FABP2 and FABP4 α-helices showed a three-dimensional overlap between lysine 21 from FABP4 and lysine 17 from hFABP2 (Fig 1B). Thus, a putative NLS in drFabp2 was mapped to lysine 17, arginine 29 and lysine 30.

**Fig 2:**
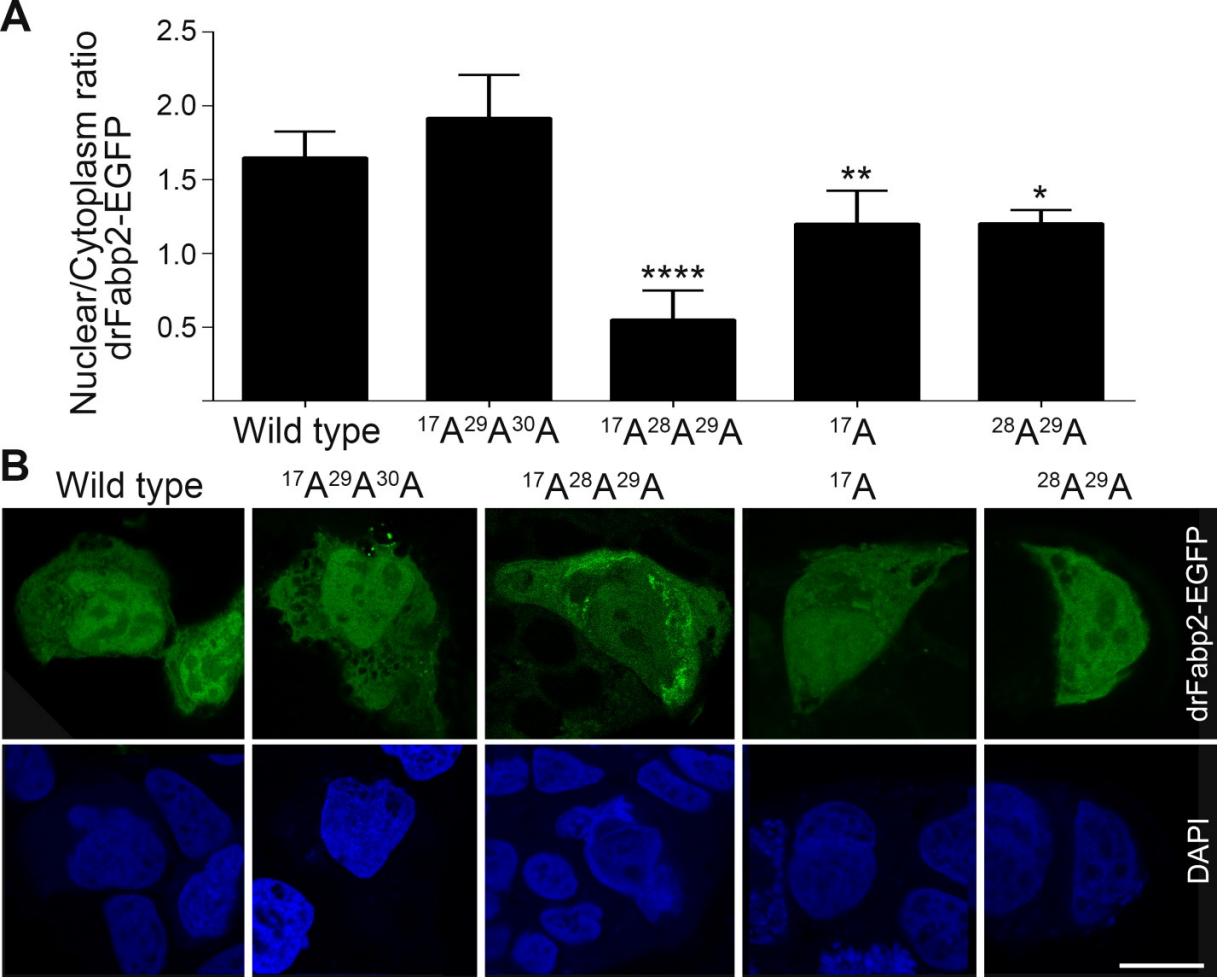
Wild-type and mutant drFabp2 subcellular localization analysis. **A)** Graphical representation of nuclear/cytoplasmic fluorescence intensity ratio (mean ± SD) of wild-type, ^17^A^29^A^30^A, ^17^A^28^A^29^A, ^17^A and ^28^A^29^A Fabp2-EGFP in Caco-2 cells. Statistical significance of each mutant with respect to wild-type Fabp2 is indicated when corresponds, **** *p < 0*.*0001*, ** *p < 0*.*01*, * p *< 0*.*05*. **B)** Representative single focal planes of Caco-2 cells transfected with wild-type, ^17^A^29^A^30^A, ^17^A^28^A^29^A, ^17^A or ^28^A^29^A Fabp2-EGFP (green). Nuclear DAPI counterstaining (blue) is also shown. Bar: 10 μm.

The amino acids of the putative NLS signal identified were then mutated to alanine and the mutant ^17^A^29^A^30^A drFabp2 EGFP-tagged protein was expressed in Caco-2 cells. Wild-type and ^17^A^29^A^30^A drFabp2-EGFP were found in the nuclei and cytoplasm of Caco-2 cells with a nucleus/cytoplasm abundance ratio indistinguishable between the two proteins (Fig 2A and 2B). Hence, the ^17^K^29^R^30^K triad does not compose an NLS in drFabp2. Taking a deeper look into α-helix II sequence, it was evident that drFabp2 contains an additional basic residue (lysine 28) that could be part of a NLS triad of basic amino acids. A new putative NLS for drFabp2, composed by lysine 17, lysine 28 and arginine 29 was mutated and the new protein was assayed for its subcellular localization in Caco-2 cells. Mutant ^17^A^28^A^29^A EGFP-tagged drFabp-2 showed a different subcellular localization than its wild-type counterpart (Fig 2A and 2B). This mutant was expressed in the cytoplasm, while its presence in the nucleus was less preponderant than for the wild-type protein (see green signal in Fig 2B, ^17^A^28^A^29^A drFabp2-EGFP image). In the case of ^17^A^28^A^29^A drFabp2, the nucleus/cytoplasm abundance ratio showed a 66% decrease compared to wild-type drFabp2 (1.65 ± 0.18 for wild type versus 0.55 ± 0.20 for ^17^A^28^A^29^A drFabp2, Fig 2A) with a p value *< 0*.*0001*. According to this, the triad ^17^K^28^K^29^R seems to constitute a functional signal to direct drFabp2 into the nucleus. In addition, basic residues located in both α-helices seem to be important. Helix I ^17^K mutant showed a nucleus/cytoplasm ratio of 1.20 ± 0.22 (Fig 2A and 2B), meanwhile a similar result was obtained when helix II basic residues ^28^K^29^R were mutated (nucleus/cytoplasmic ratio = 1.20 ± 0.09, Fig 2A and 2B). For both helix mutants the nucleus/cytoplasm abundance ratio showed a 27% decrease compared to wild-type drFabp2-EGFP, with *p < 0*.*01* for the ^17^A mutant and *p < 0*.*05* for the ^28^A^29^A mutant, respectively (Fig 2A). It is noteworthy that both α-helices seem to contribute cooperatively to the nuclear localization of drFabp2, since the mutation in one helix is not enough to reduce the nuclear localization to the levels observed in the mutant of both helices.

**Fig 3:**
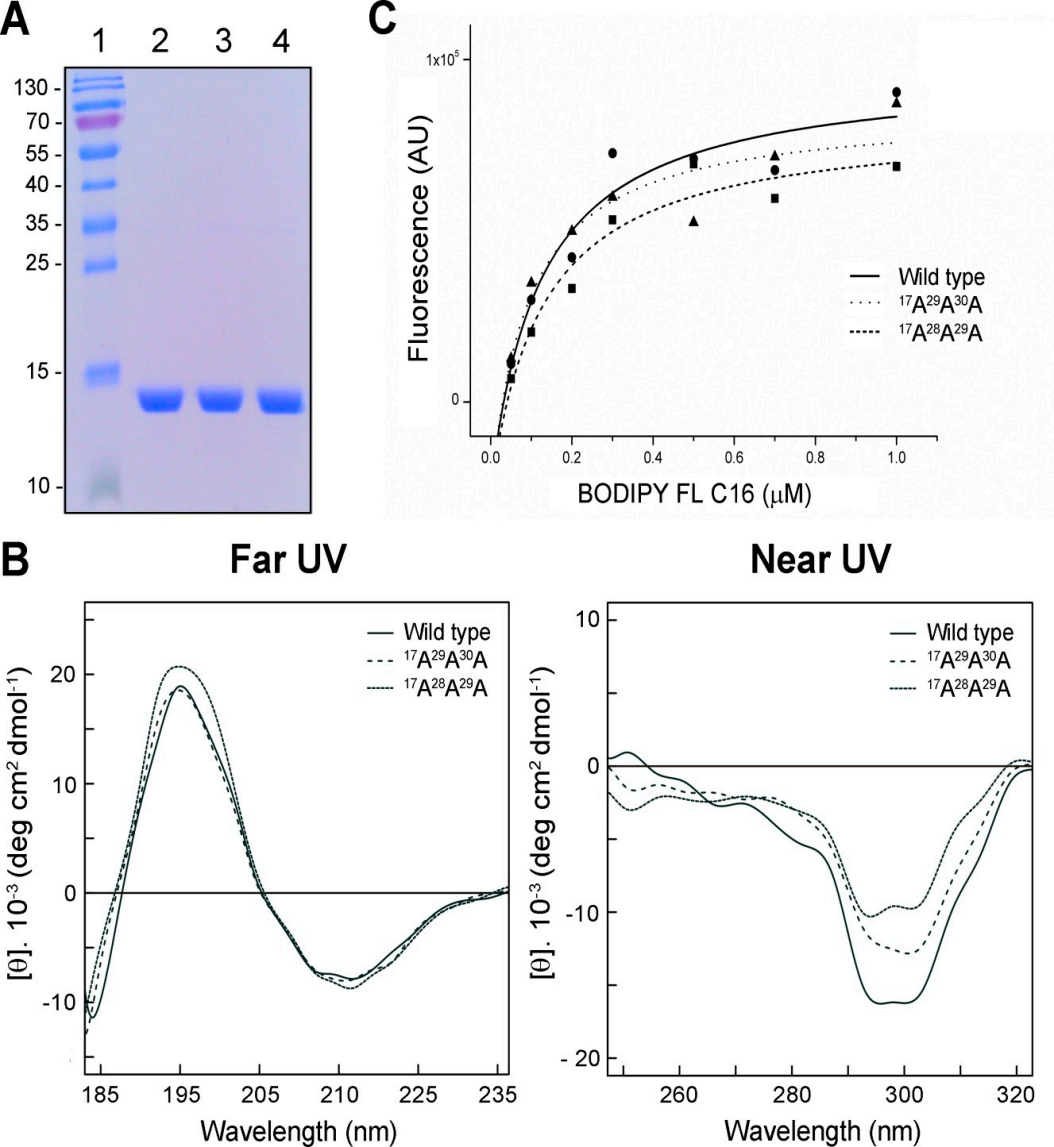
Wild-type and mutant drFabp2 structural analysis. **A)** SDS-PAGE of purified wild-type (lane 2), ^17^A^29^A^30^A (lane 3) and ^17^A^28^A^29^A (lane 4) drFabp2. The molecular weight of the proteins is indicated in kDa in Standard (lane 1). **B)** Circular dichroism analysis of wild-type, ^17^A^29^A^30^A and ^17^A^28^A^29^A drFabp2 in the far- (185–235 nm) and near- (250–320 nm) UV spectra. **C)** wild-type, ^17^A^29^A^30^A and ^17^A^28^A^29^R drFabp2-BODIPY FL C16 binding affinity determination. Fluorescence intensity values were recorded at 510 nm (λex) and 530 nm (λem) using 0.5 μM of each protein.

In order to confirm that the ^17^A^29^A^30^A and ^17^A^28^A^29^A drFabp2 mutants have the same structure that the wild-type one, recombinant proteins were expressed in *E*. *coli* BL21 (DE3) under the same conditions and purified using the same protocol. Electrophoretic behavior, circular dichroism and binding assays were performed. The three proteins showed the same behavior in salt fractionation, chromatographic procedures, and yielded similar quantities of protein by mL of culture (wild type: 1.04 mg/mL; ^17^A^29^A^30^A: 0.73mg/mL; ^17^A^28^A^29^A: 1.54 mg/mL). Electrophoretic behavior of the wild-type and mutated proteins was unchanged (Fig 3A). The secondary and tertiary structure of the wild-type and drFabp2 mutants was analyzed by far-and near-UV CD, respectively. The obtained data showed that the secondary structures of all forms of the protein were identical within the experimental error of the technique. Typical β-sheet signal was observed at 196 nm and 216 nm for wild-type, ^17^A^29^A^30^A and ^17^A^28^A^29^A drFabp2 (Fig 3B). These signals are in accordance with previous reports [43, 44]. Concerning tertiary structure, it follows from obtained data that the structures are highly similar, with similar minimums in the spectrum (Fig 3B). Dissociation constants calculated from titration curves indicated that all three proteins maintained similar ligand-binding capabilities (Fig 3C), with Kd values of 0.160 μM for wild-type, 0.129 μM for ^17^A^29^A^30^, and 0.112 μM for ^17^A^28^A^29^A This data is in the same order as previous reports [13].

**Fig 4:**
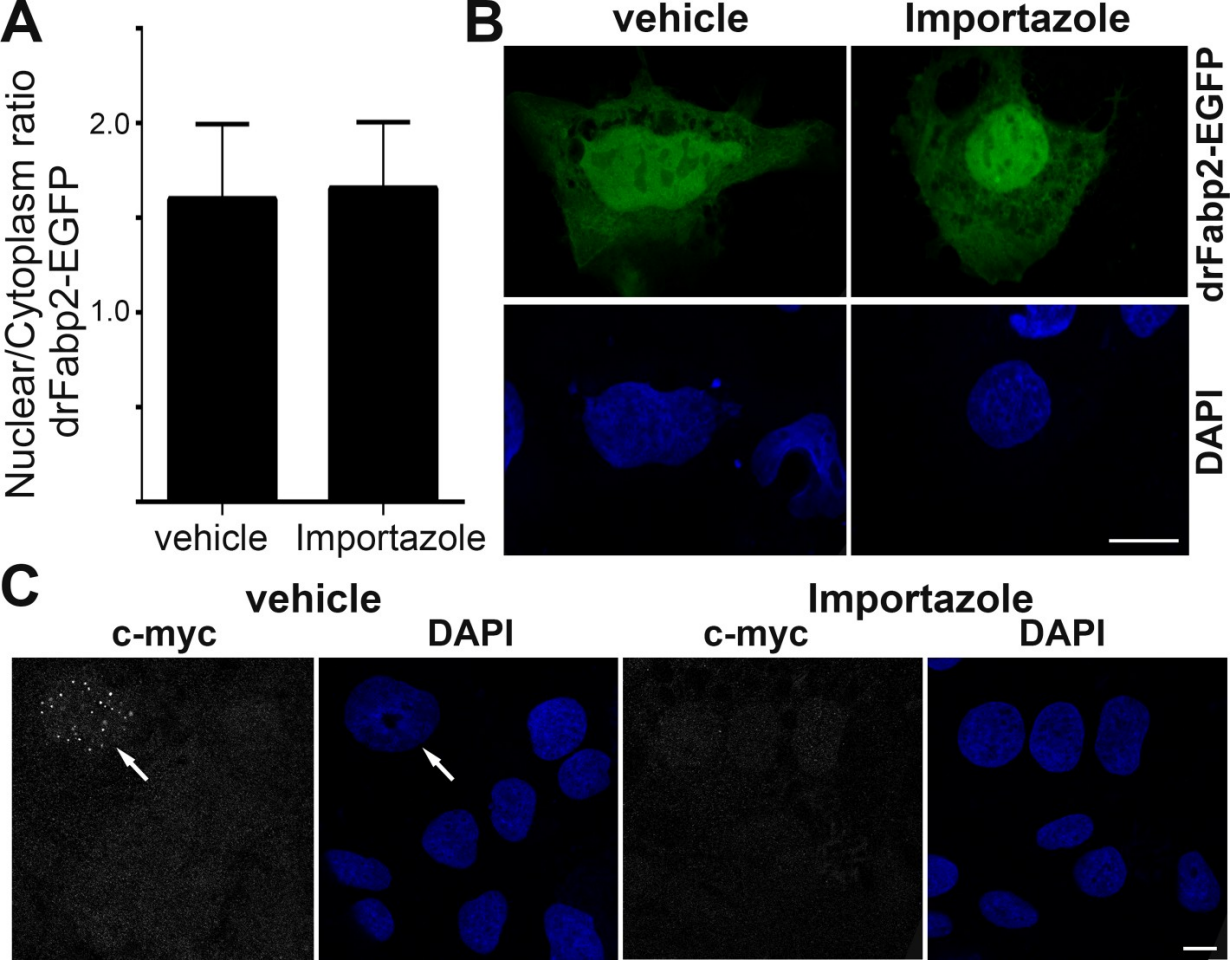
Wild-type drFABP2 nuclear import is independent of β-importin activity. **A)** Graphical representation of nuclear/cytoplasmic fluorescence intensity ratio (mean ± SD) of wild type Fabp2-EGFP in Caco-2 cells cultured in media with vehicle or in media with 40 μM Importazole. **B)** Representative single focal planes of Caco-2 cells transfected with wild type Fabp2-EGFP (green) cultured in media with vehicle or in media with 40 μM Importazole. Nuclei counterstaining with DAPI is shown in blue. **C)** Representative single focal planes of anti-c-Myc signal (gray) in Caco-2 cells cultured in media with vehicle or in media with 40 μM Importazole. Nuclei counterstaining with DAPI is shown in blue. Arrow points to a c-Myc positive nucleus. Bar: 10 μm.

To assess if the classical nuclear import mechanism is involved in drFabp2 transport, we monitored its nuclear presence using the β-importin inhibitor Importazole. All cells positive for drFabp2-EGFP showed a nuclear localization of the protein in vehicle- and Importazole-treated conditions. The nuclear/cytoplasmic ratio in the vehicle-treated cells was indistinguishable from that of the Importazole-treated cells (1.60 ± 0.40 versus 1.65 ± 0.35, respectively, *p = 0*.*5917*, Fig 4A and 4B). This suggests that importin mediated transport would not be responsible for the transfer of Fabp2 from the cytoplasm to the nucleus. As a positive control, nuclear c-Myc signal was evaluated. While 5.31 ± 1.13% of vehicle-treated cells showed c-Myc nuclear punctate staining, none of the Importazole-treated cells showed nuclear localization of the transcriptional factor, confirming the β-importin mediated-transport inhibition by Importazole in the assayed conditions (Fig 4C).

**Fig 5:**
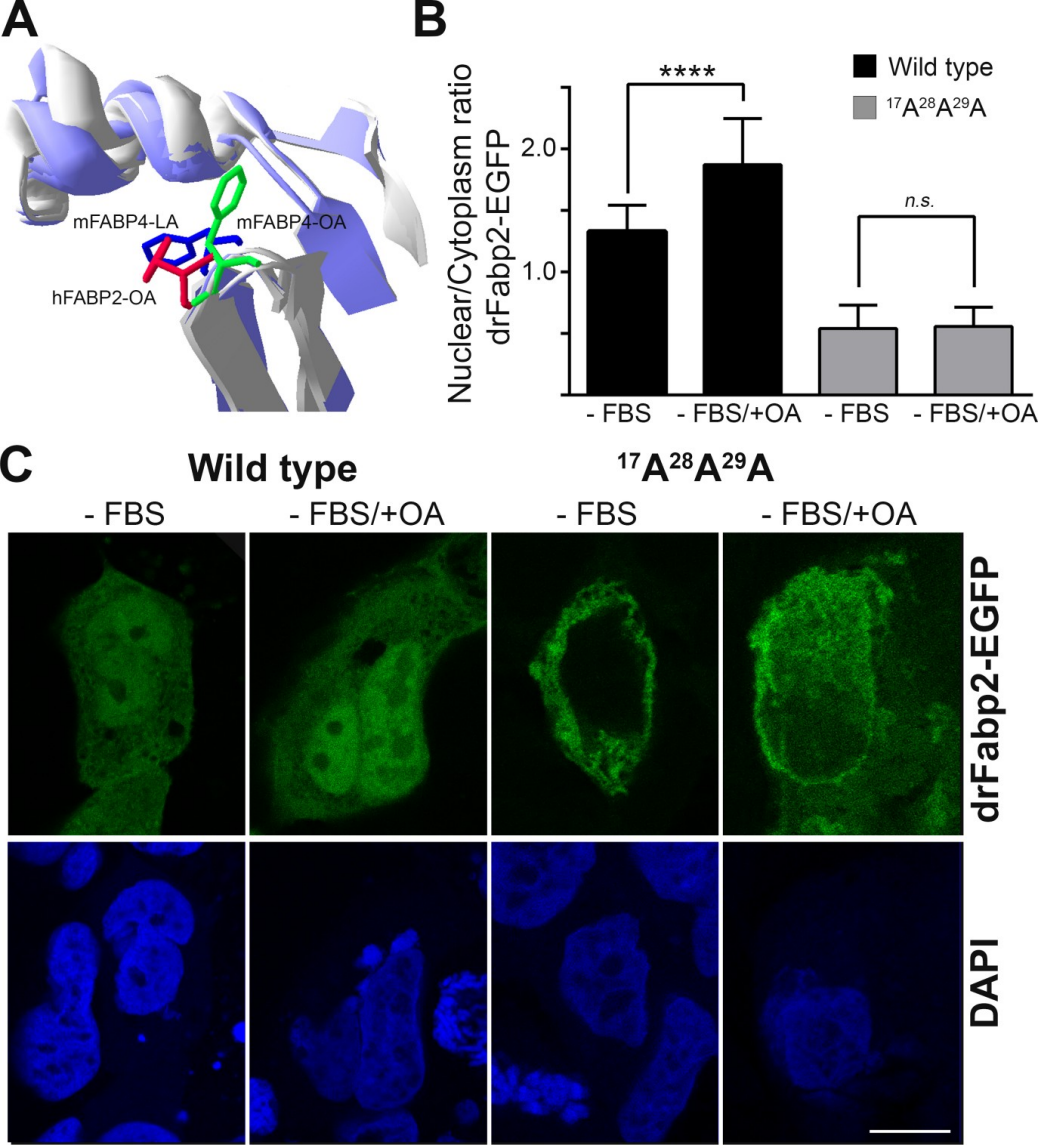
Wild-type and mutant drFabp2 ligand-dependent activation of nuclear traslocation. **A)** Superimposed structures of the α-helical regions of mFABP4 bound to linolenic acid (mFABP4-LA, PDB ID: 2Q9S), mFABP4 bound to oleic acid (mFABP4-OA, PDB ID: 1LID) and hFABP2 bound to oleic acid (hFABP2-OA, PDB ID: 2MO5) α-helical region. The side chain of the Phe residue implicated in ligand-dependent response to ligands is shown for FABP4 bound to linolenic acid (blue), mFABP4 bound to oleic acid (green) and hFABP2 bound to oleic acid (red). **B)** Graphical representation of nuclear/cytoplasmic fluorescence intensity relationship (mean ± SD) of wild- type and ^17^A^28^A^29^A drFabp2-EGFP in Caco-2 cells cultured in media without FBS (-FBS) or in media without FBS and with oleic acid (-FBS/+OA). Statistical significance is indicated, **** *p < 0*.*0001*, n.s. not significant. **C)** Representative single focal planes of Caco-2 cells transfected with wild- type or ^17^A^28^A^29^A drFabp2-EGFP (green) cultured in media without FBS (-FBS) or in media without FBS and with oleic acid (-FBS/+OA). Nuclei counterstaining with DAPI is shown in blue. Bar: 10 μm.

FABPs import to the nucleus has been proposed to be dependent on the protein binding to activating or inactivating ligands. Linoleic acid (LA) is an mFABP4 activating ligand, while oleic acid (OA) is not. Binding of mFABP4 to an activating ligand changes the position of a phenylalanine in the loop between β-sheet C and D, which in turn results in a subtle shift in the α-helical domain, proposed to be responsible for NLS exposure [39]. A structural analysis of hFABP2 conjugated to OA, compared to holo-mFABP4, showed that hFABP2 Phenylalanine 56/57 from loop C-D occupies the same position as in mFABP4-LA (Fig 5A). Quantification of nuclear/cytoplasm ratio of wild-type drFabp2-EGFP in the presence or absence of OA showed that a nuclear increment of the protein takes place when the fatty acid is present (1.87 ± 0.37 with OA versus 1.33 ± 0.21 without OA, *p < 0*.*0001*, Fig 5B and 5C). OA-responsive subcellular localization was not observed in the ^17^A^28^A^29^A mutant. The nuclear/cytoplasm ratio of this mutant remains unchanged in the absence or presence of the fatty acid (0.56 ± 0.16 without OA versus 0.54 ± 1.9 with OA, not significant), confirming that ^17^K^28^K^29^R is responsible for nuclear localization in drFabp2.

## 4. Discussion

A great deal of work has been done concerning the capture and intracellular transport of fatty acids, with FABPs being good candidates for this role. These are small soluble proteins that reversibly bind FAs, trafficking them to different subcellular compartments. In addition, they can uptake FA from model phospholipids vesicles and transfer their cargo to membranes [7, 45–49].

The nuclear localization of many FABP family members has led to suggest a role in nuclear lipid metabolism, as well as in the regulation of gene expression [32, 50, 51]. It has been proposed that FABPs transfer their cargo to activate peroxisome-proliferator-activated-receptors (PPARs), acting as gene transcription modulators [33–35, 38, 52, 53]. Recently, it was reported that *Danio rerio* Fabp1 and Fabp2 were localized in enterocyte nuclei and that they have the potential to channel dietary fatty acids into this compartment [30].

While classic NLS are composed of conserved linear basic amino acids sequences, FABPs NLS have been described as a three-dimensional fold of three basic amino acids [35, 38, 54, 55]. The signal is composed by a basic residue on helix I and two basic residues on helix II. However, the sequence alignment of hFABP2 and drFabp2 with previously reported signals does not show a perfect match. Furthermore, drFabp2 has three consecutive basic residues (lysine 28, arginine 29, lysine 30) on helix II, absent in other proteins of the family. Only residues ^17^K, ^29^R and ^30^K overlap on the 3D-solved structures of human FABP2 and FABP4. Surprisingly, these amino acids do not conform an NLS in drFabp2. We demonstrated the triad ^17^K, ^28^K and ^29^R to be responsible for Fabp2 cytoplasm-to-nucleus translocation, an unexpected result since the ^28^K residue on human solved FABP2 has a structurally opposite position when compared to that of the reported signals.

The role of the helical region of FABPs has been extensively studied. Helices I and II also conform to the portal entrance of ligands. The movements of atoms of this region and interactions with the carboxylate head of the ligand could be critical steps in directing it to the interior of the protein [56]. Basic residues present in this region are involved in membrane interactions and play a critical role in the collisional mechanism of fatty acid transfer from FABP2 to phospholipid membranes [57–60]. FABPs helical domain is also responsible for protein-protein interaction, particularly with Cgi-58 [61]. In addition, we here showed that basic residues in helices I and II are indispensable for nuclear transport of drFabp2. For the conventional NLS, basic residues are recognized by the α/β-importin heterodimer that also mediates the docking to the nuclear pore complex, followed by the protein complex translocation to the nucleus [62]. In a *in silico* study, Amber-Vitos *et al* [63] indicates that FABP4 interacts with α-importin, proposing the classical nuclear import mechanism as responsible for FABP transport. In spite of the scenario proposed by Amber-Vitos *et al*, our β-importin inhibition experiment indicates that the classical nuclear transport mechanism would not be involved in the Fabp2 nuclear translocation, and therefore another not diffusional mechanism should be responsible for its transfer [64]. Our results are consistent with a previous report indicating that FABP2 does not interact with α/β-importin heterodimer *in vitro* [36]. Elucidating the exact nuclear import mechanism of drFabp2 protein will be the subject of our future efforts

In conclusion, using molecular techniques and microscopy observations, we show that drFabp2 has a structural non-classical nuclear localization signal conformed by the basic triad ^17^K^28^R^29^K, and that this signal could be activated by oleic acid.

## Supporting information

S1 Fig (JPG)
