# Supplementary figures and images for: Identification of a non-classical three-dimensional nuclear localization signal in the intestinal fatty acid binding protein
Source: PLoS One. 2020 Nov 12;15(11):e0242312. doi: 10.1371/journal.pone.0242312 (PMC7660557; doi:10.1371/journal.pone.0242312)

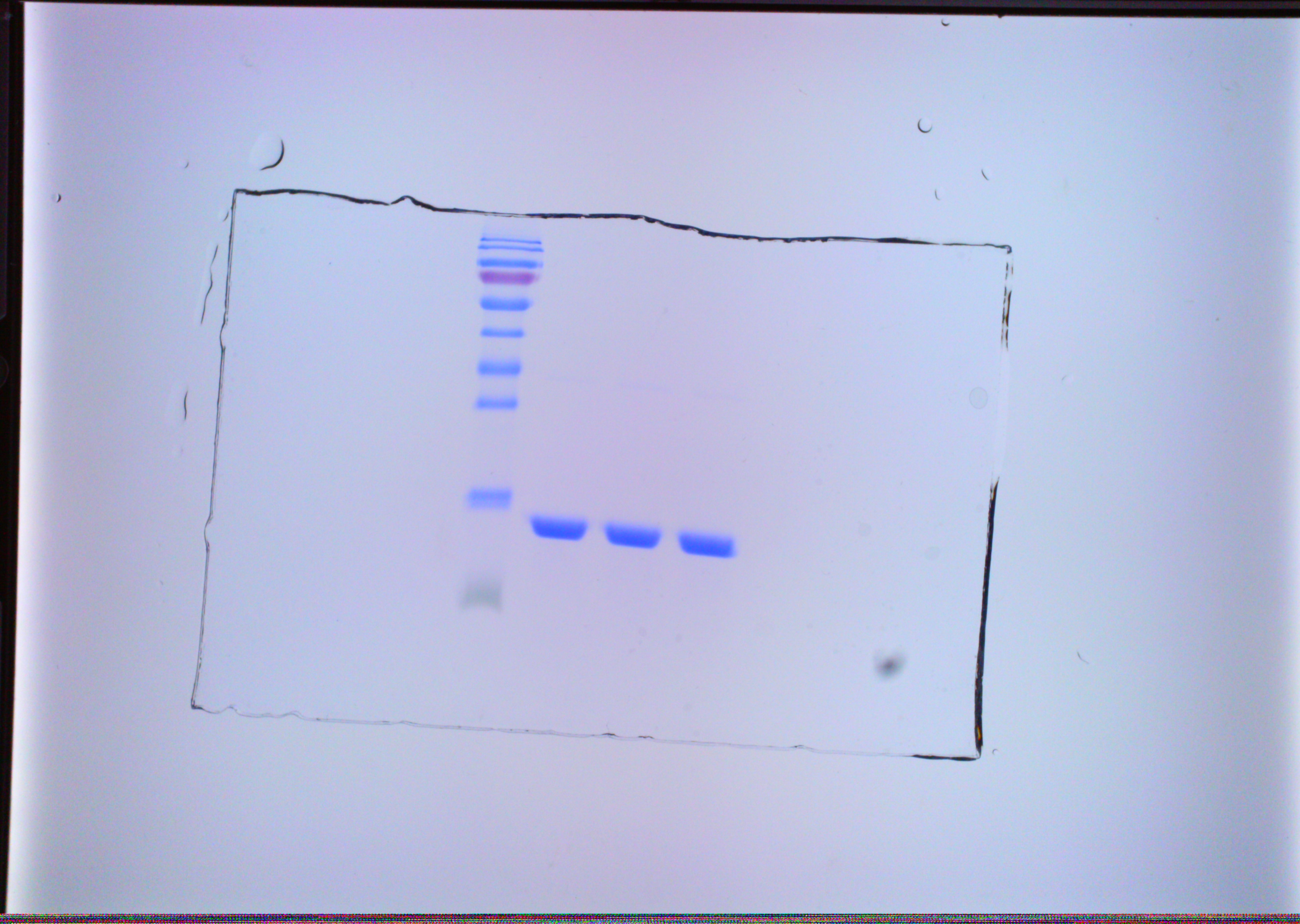

Supplement: S1 Fig — (JPG) [file pone.0242312.s001.JPG]
